# Single high-dose intravenous injection of Wharton’s jelly-derived mesenchymal stem cell exerts protective effects in a rat model of metabolic syndrome

**DOI:** 10.1186/s13287-024-03769-2

**Published:** 2024-06-05

**Authors:** Alvin Man Lung Chan, Angela Min Hwei Ng, Mohd Heikal Mohd Yunus, Ruszymah Hj Idrus, Jia Xian Law, Muhammad Dain Yazid, Kok-Yong Chin, Mohd Rafizul Mohd Yusof, See Nguan Ng, Benson Koh, Yogeswaran Lokanathan

**Affiliations:** 1 Centre for Tissue Engineering and Regenerative Medicine, Faculty of Medicine, Universiti Kebangsaan Malaysia, 56000 Cheras, Kuala Lumpur, Malaysia; 2 Department of Physiology, Faculty of Medicine, Universiti Kebangsaan Malaysia, 56000 Kuala Lumpur, Malaysia; 3 Department of Pharmacology, Faculty of Medicine, Universiti Kebangsaan Malaysia, 56000 Kuala Lumpur, Malaysia; 4 Department of Parasitology and Medical Entomology, Faculty of Medicine, Universiti Kebangsaan Malaysia, 56000 Kuala Lumpur, Malaysia; 5 Ming Medical Sdn Bhd, D3-3 (2nd Floor), Block D3 Dana 1 Commercial Centre, Jalan PJU 1a/46, 47301 Petaling Jaya, Selangor, Malaysia

**Keywords:** Cell therapy, Metabolic syndrome, Mesenchymal stem cell, Syndrome X Wharton’s jelly

## Abstract

**Background:**

Metabolic syndrome (MetS) is a significant epidemiological problem worldwide. It is a pre-morbid, chronic and low-grade inflammatory disorder that precedes many chronic diseases. Wharton’s jelly-derived mesenchymal stem cells (WJ-MSCs) could be used to treat MetS because they express high regenerative capacity, strong immunomodulatory properties and allogeneic biocompatibility. This study aims to investigate WJ-MSCs as a therapy against MetS in a rat model.

**Methods:**

Twenty-four animals were fed with high-fat high-fructose (HFHF) diet ad libitum. After 16 weeks, the animals were randomised into treatment groups (n = 8/group) and received a single intravenous administration of vehicle, that is, 3 × 10^6^ cells/kg or 10 × 10^6^ cells/kg of WJ-MSCs. A healthy animal group (n = 6) fed with a normal diet received the same vehicle as the control (CTRL). All animals were periodically assessed (every 4 weeks) for physical measurements, serum biochemistry, glucose tolerance test, cardiovascular function test and whole-body composition. Post-euthanasia, organs were weighed and processed for histopathology. Serum was collected for C-reactive protein and inflammatory cytokine assay.

**Results:**

The results between HFHF-treated groups and healthy or HFHF-CTRL did not achieve statistical significance (α = 0.05). The effects of WJ-MSCs were masked by the manifestation of different disease subclusters and continuous supplementation of HFHF diet. Based on secondary analysis, WJ-MSCs had major implications in improving cardiopulmonary morbidities. The lungs, liver and heart show significantly better histopathology in the WJ-MSC-treated groups than in the untreated CTRL group. The cells produced a dose-dependent effect (high dose lasted until week 8) in preventing further metabolic decay in MetS animals.

**Conclusions:**

The establishment of safety and therapeutic proof-of-concept encourages further studies by improving the current therapeutic model.

## Background

Metabolic syndrome (MetS) is a cluster of abnormal physiological conditions, often linked by genetic and environmental disruption. MetS is associated with many chronic diseases such as obesity, type 2 diabetes mellitus, cardiovascular diseases (CVDs), non-alcoholic fatty liver disease and even osteoporosis [1–5]. Operationally, MetS occurs when three of the five hallmarks, namely, increased body weight or abdominal circumference, hyperglycaemia (increased blood sugar levels), hypertension (high blood pressure), hypertriglyceridemia and dyslipidaemia (disproportionate cholesterol levels), are present [6]. Having these traits leads to increased mortality, morbidity and healthcare costs if left untreated [7, 8]. Despite the availability of off-the-shelf pharmaceutics to manage conditions such as high cholesterol, hypertension or hyperglycaemia [9–12], they only offer temporary relief and do not address the root cause [13, 14]. In response, major efforts have been taken in the field of regenerative medicine. Despite previous controversies, stem cell therapies have surged in the past decade. Thorough exploration and proper scientific communication have been delivered on its safety, which is a major hurdle for cell-based therapy [15–18].

This study aimed to establish the safety and efficacy of Wharton’s jelly-derived mesenchymal stem cell (WJ-MSC) for the treatment of MetS. Owing to potent MSCs, the previously discarded umbilical cord has been repurposed into a biological remedy [19, 20]. Whilst bone marrow-derived MSCs (BM-MSCs) were thoroughly studied and said to be the gold standard for sourcing MSCs, the latest review has described WJ-MSCs to be equally competent, if not, superior [21–23]. MSCs from either source share a similar profile of high regenerative capacity and general immune privilege from allogeneic transplantation. As per the ISCT classification, the cells were also able to undergo trilineage differentiation (adipocyte, chondrocyte and osteoblast) when grown in a specific medium. Besides, MSCs participate in intercellular signalling via the secretion of active metabolites. These extracellular vesicles (canonically as ‘*exosomes*’) can stimulate regeneration in injured areas by modulating excess inflammation and supplementing growth factors [24]. An additional benefit of WJ-MSCs that remains debated is their homing and migratory properties [25]. The cells and their derivatives possess chemotactic proteins and/or receptors on their surface corresponding to the inflammatory signals or apoptotic bodies [26, 27]. This feature enables migration into difficult-to-reach areas. Considering that MSCs and their paracrine effects influence multiple biological systems, they have the potential to alleviate the symptoms of MetS.

Following up on the safety study on WJ-MSCs, this paper aimed to establish the benefits of high or low doses of intravenously (IV)-administered WJ-MSCs versus no treatment for MetS in a similar animal model. The treatment was hypothesised to behave in a dose-dependent manner, where a higher dose could elicit a larger and prolonged effect. The results will be extrapolated and modelled into larger organisms for its intended human applications in the future.

## Materials and methods

### Culture of WJ-MSCs

Umbilical cord was procured with consent from maternal volunteers undergoing elective Caesarean section at Universiti Kebangsaan Malaysia Medical Centre, Malaysia. The WJ-MSCs were pooled from three donors and qualified via characterisation, fibroblast-like cell morphology and attachment to plastic surfaces, expression of positive (≥ 95%; CD44, CD73, CD90 and CD105) and negative markers (≤ 3%; CD11b, CD19, CD34, CD45 and HLA-DR) by using a Human MSC Analysis Kit (BD Bioscience, USA) and trilineage differentiation using a manufacturer’s kit (Gibco, USA). The same source of cryopreserved cells was used in the safety study to ensure accurate representation of data for pre-clinical assessment of this cell therapy [28]. Briefly, the Passage 5 WJ-MSCs were thawed from cryo-storage and cultured into flasks the day before. After overnight culture, the attached cells were harvested, enumerated and prepared into syringes at 3 × 10^6^ cells/kg [high-fat high-fructose (HFHF)-low dose (LD)] or 10 × 10^6^ cells/kg body weight (BW, HFHF-high dose (HD)] for intravenous infusion.

### Animals

Thirty male specific pathogen-free, 12-week-old Sprague Dawley rats were purchased from the Malaysia Institute of Pharmaceuticals & Nutraceuticals, National Institute of Biotechnology Malaysia (Penang, Malaysia). The animals were housed individually in closed ventilated cages via the Biobubble system (Allentown Inc., USA) located at the Centre for Tissue Engineering and Regenerative Medicine, Universiti Kebangsaan Malaysia. Additionally, the room was set at a temperature of 22 °C with a 12 h light-and-dark cycle. The rodents were given the standard lab chow (Altromin 1314; Lage, Germany) and autoclaved tap water ad libitum during the 2-week acclimatisation. The conduct and findings of the papers are reported in accordance to the ARRIVE Guidelines 2.0, ensuring rigorous and transparent reporting of our animal-based research.

### Animal study plan

#### Induction of metabolic syndrome (from week − 16 to Week 0)

After acclimatisation, the animals were randomly divided into two diet groups. The HFHF diet group (n = 24) was fed with high-fat pellet (Altromin C 1090–70; Lage, Germany) and 30% w/v of crystalline fructose (Aurora Industry Co. Ltd., China) dissolved in autoclaved tap water. The remaining animals (n = 6) were assigned to the normal diet (ND) group, which received standard lab chow (Gold Coin, Malaysia) and autoclaved tap water. Both diets were given ad libitum for 16 weeks. The success of MetS induction was determined by the presence of three out of the five MetS hallmarks; increased abdominal circumference, hypertension, hyperglycaemia, hypertriglyceridemia, and dyslipidaemia. Each of these physiological attributes was determined via measurable tests listed in Sect.  5.6.

#### Treatment of MetS with WJ-MSC (From Week 0 to Week 12)

After the animal model of MetS was established, the HFHF-diet animals were randomly assigned (n = 8 per group) to three treatment groups that received either 0.9% sodium chloride solution as blank or control (CTRL), 3 × 10^6^ cells/kg BW as LD or 10 × 10^6^ cells/kg BW as HD. The ND group received a blank solution. The treatment proceeded for 12 weeks as previously designed in the safety assessment study [28]. The animals were subjected to 12 weeks of periodic tests performed intermittently (every 4 weeks), which involved physical measurements, serum biochemistry, cardiovascular function test and whole-body composition analysis. Physical measurements and cardiovascular function tests were performed with minimal restraint. Blood collection via periorbital sinus and whole-body composition analysis required anaesthesia using 0.1 mL/kg BW of ketamine:xylazine (1:10) (K-X) cocktail (Troy Laboratories, Australia). Signs of morbidity and mortality were observed intermittently throughout the study. After 12 weeks, the animals were euthanised by chemical overdose via intraperitoneal (IP) administration of 2 mL pentobarbital (200 mg/mL, Vetoquinol, UK). Necropsy and histological staining of organs were performed thereafter.

### Parameters for animal study

#### Signs of morbidity and mortality

Physical observation of animals was performed intermittently, whenever possible. Their external and observable physiological states were adopted from ‘*Endpoint guidelines for animal use protocols*’ (2018), drafted by the University of Maryland (School of Medicine) and approved by the Institutional Animal Care and Use Committee (IACUC). Human endpoints were adopted and paraphrased from their protocol as described below:

- *Severe weight loss from anorexia and/or dehydration.*
- *Dyspnoea (laboured breathing, hyperventilation and abdominal distension).*
- *Prolonged hypothermia or hyperthermia (palpable temperature).*
- *Stress and/or poor grooming (rough stained coat and porphyrin built around nose and eyes).*
- *Lethargy, hunched posture and inability to rise or ambulate.*
- *Poor reflex or irresponsiveness to external stimuli.*
- *Tumour growth.*

#### Physical measurement

The animals were lightly restrained using plastic decapicones without anaesthesia or sedatives. The BW, body length (nose to anus) and abdominal circumference (Abd. Circ.) were recorded. The body mass index (BMI) of the animals was calculated by the ratio of body weight to body length squared. Food intake was measured through the weight (g) of diet consumed per week. Water intake was calculated by volume (mL) consumed per week on the basis of the changed weight (g) of the bottled water. Physical measurements were taken weekly but presented in averages of study periods: weeks 0, 4, 8 and 12.

#### Blood serum analysis

Similarly, the animals were bled at weeks 0, 4, 8 and 12 via the tail vein after IP anaesthesia of the K-X cocktail. Blood samples were collected using clot-activator tubes (BD Biosciences, USA) and allowed to clot at room temperature for 15 min. Serum was collected via centrifugation at 3000 rcf for 10 min and subsequently stored in the freezer (− 20 °C) until needed. Serum biochemistry was conducted in the Haematology Laboratory located at the Veterinary Laboratory Service Unit, Universiti Putra Malaysia, Malaysia, to measure the concentration of aspartate aminotransferase (AST), alanine aminotransferase (ALT), creatinine (CREAT), cholesterol (CHOL), high-density lipoprotein (HDL), low-density lipoprotein (LDL), triglyceride (TGL) and serum glucose (GLUC).

#### Oral glucose tolerance test (OGTT)

Before the day of the experiment, the animals were fasted overnight with ad libitum access to autoclaved tap water only. On the following day, the fasting blood glucose (FBG) levels were measured using an ACCU-Check Performa glucometer (Roche Diagnostic, USA). After baseline measurement, the animals were given 20% dextrose solution (2 µL/g BW) via oral gavage. Afterwards, they were bled at intervals of 30, 60 and 120 min. In addition to weekly comparison of the different treatment groups, the area under the curve (AUC) was calculated and plotted as a summary of GLUC levels at different study periods.

#### Cardiovascular function test

Diastolic blood pressure (DBP), systolic blood pressure (SBP), mean arterial pressure (MAP), blood flow and volume and heart rate were measured using a non-invasive tail-cuff system (CODA, Kent Scientific Corporation, USA). The animals were restrained using plastic decapicones without anaesthesia and briefly warmed on a far-infrared warming platform (Kent Scientific Corporation, USA) before measurements were taken. TCODA software was programmed to measure five acclimatisation cycles and 10 experimental cycles, with each cycle spaced between 30 s to allow recovery of blood flow. Data were exported into Microsoft Excel 2019 MSO 64-bit (Microsoft Corp., USA) for further analysis.

#### Whole-body composition

The animals were anesthetised and laid in an anatomically prone position with their head, tail and limbs straightened outwards. The fat mass, lean mass, percentage of body fat, bone mineral content (BMC) and bone mineral density (BMD) were measured using Small Animal Analysis Software in a dual-energy X-ray absorptiometry machine (Hologic QDR-1000 System, Hologic Inc., USA). The short-term in-vivo coefficient of variation for whole-body BMD was 1.2% for this machine [29].

#### Necropsy

During the end of study at week 12, the animals were anaesthetised using the K-X cocktail and then euthanised via chemical overdose using 2 mL of pentobarbital sodium. When the animals were determined to be no longer conscious via toe-pinch response method, blood was drained from the animals’ carcass via cardiac puncture. Blood serum was collected and stored for supplementary experiments, excluding the final serum biochemistry test. The animals were dissected at the abdomen to reveal its subcutaneous adipose layer. Fat was collected via scalpel shaving. Then, the animals were dissected to reveal the thoracic and abdominal cavity. All the organs, including the heart, lung, liver, spleen, kidneys, pancreas and gut (large intestine); the bone (right femur); and the visceral fat were excised. Images were taken at all stages and during the harvesting of organs. The mass of the organs was recorded immediately using a weighing scale. The relative weight of the organs was calculated as below:

$$Relative \, weight \, \left( g \right) \, = \, \left[ {mass \, of \, organ \, \left( g \right)/animal^{\prime}s \, body \, weight \, \left( g \right)} \right] \, \times \, 100$$

#### Histological staining of tissue sections

The harvested lung, liver, spleen and kidneys were preserved in a 4% paraformaldehyde solution before being embedded in paraffin. Sections were cut using a microtome (Leica Biosystems, Wetzlar, Germany), deparaffined with xylene and stained with haematoxylin and eosin (H&E). The stained section was observed under a light microscope by a blinded histopathologist to check for microanatomical conditions of the organs. The scoring was described as healthy or inflamed (minor, moderate or severe).

#### ELISA

The serum concentration of C-reactive protein (CRP) was analysed using an ELISA kit purchased from Raybiotech (Georgia, USA). Serum dilutions and the assay procedure was performed in accordance with the manufacturer’s protocol.

#### Multiplex ELISA cytokine array

The Quantibody rat inflammation array was purchased from Raybiotech (Georgia, USA), and 96-well plates were designed to detect 10 inflammatory-associated cytokines: interferon-gamma (IFN-γ), interleukins (IL-10, IL-13, IL-1α, IL-1β, IL-2, IL-4 and IL-6), monocyte chemoattractant protein-1 (MCP-1) and tumour necrosis factor-alpha (TNF-α). Serum dilutions and the assay procedure were performed following the manufacturer’s protocol.

### Statistical analysis

All sample sizes were determined by Mead’s resource equation. All statistical analysis was performed using GraphPad software (GraphPad Prism version 8.4.3, California, USA). The quantitative data or graphical results were presented as mean ± standard deviation (SD). Comparisons between the four treatment groups for physical measurement, serum biochemistry, cardiovascular function and whole-body composition were conducted through mixed-design ANOVA with Geisserg–Greenhouse correction. Time-fixed inter-group analysis was calculated via Holm Sidak’s post-hoc test (*P* < 0.05). End-point comparisons, such as the relative weight of the organs, were performed using one-way ANOVA followed by Holm Sidak’s post-hoc test (*P* < 0.05). Statistical significance was set at *P* < 0.05.

## Results

### HFHF diet successfully induced MetS in animal model

After 16 weeks of the diet regime, the animals successfully achieved at least three of the five MetS hallmarks. The shared characteristics amongst all the treated animals (n = 24) were hypertension (increased SBP, DBP or MAP), hyperglycaemia (increased serum GLUC) and dyslipidaemia (reduced HDL cholesterol). All animals were screened individually and compared with the healthy CTRL animals (Appendix 1). Five animals achieved the minimum score of 3, whereas most animals achieved a higher score of 4 (n = 10) or 5 (n = 9). Furthermore, the animals showed signs of MetS-related stress, as shown in Table 1. All HFHF animals had significant dyspnoea and secretion of porphyrin, indicating physical and metabolic stress. Some animals experienced severe weight loss, low food consumption, lethargy and poor response to stimuli.

**Table 1:** Signs of morbidity and mortality of animals throughout the study

Symptoms	Study Period			
	Week 0	Week 4	Week 8	Week 12
Anorexia, weight loss and/or dehydration	HFHF only (n = 2/group)	HFHF only (n = 2/group)	HFHF only (n = 2/group)	HFHF only (n = 2/group)
Dyspnea	HFHF only (n = 8/group)	HFHF only (n = 8/group)	HFHF only (n = 8/group)	HFHF only (n = 8/group))
Prolonged hypothermia or hyperthermia	–	–	–	–
Stress and/or poor grooming	HFHF only (n = 8/group)	HFHF only (n = 8/group)	HFHF only (n = 8/group)	HFHF only (n = 8/group))
Lethargy, hunched posture and inability to rise or ambulate	HFHF only (n = 2/group)	HFHF only (n = 2/group)	HFHF only (n = 2/group)	HFHF only (n = 2/group)
Poor reflex or irresponsiveness to external stimuli	HFHF only (n = 2/group)	HFHF only (n = 2/group)	HFHF only (n = 2/group)	HFHF only (n = 2/group)
Tumor growth	–	–	–	–

Symbols: (–) indicate no observable symptoms

### WJ-MSC was unable to resolve MetS disorders in animal model

**Fig. 1:**
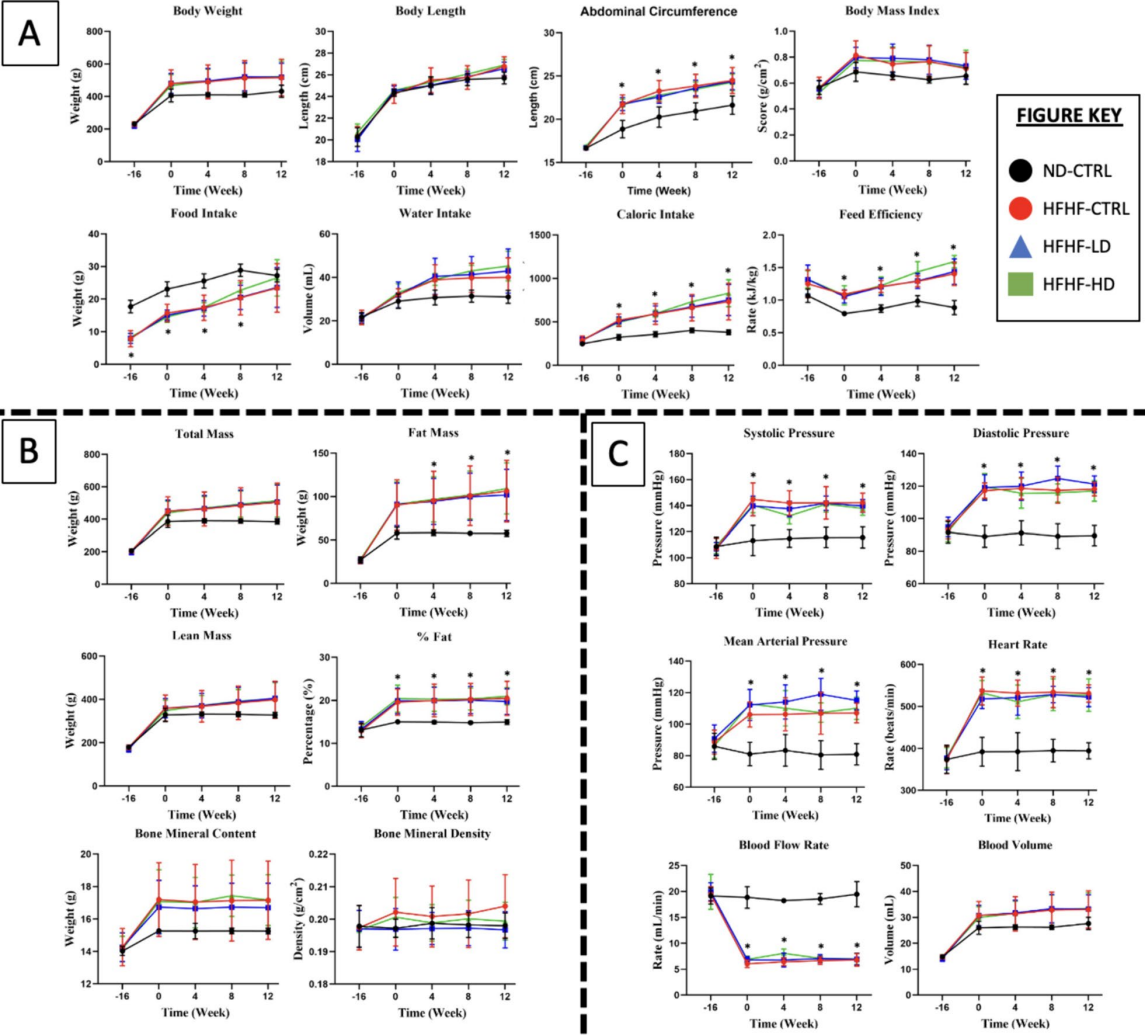
**A** Physical measurement, **B** whole-body composition and **C** cardiovascular function tests performed at weeks − 16, 0, 4, 8 and 12 for treatment (n = 8) and control (CTRL) animals (n = 6) in HFHF diet or ND diet groups. Symbols: (#) indicates that the HFHF group’s data are statistically significantly different (*P* < 0.05) compared with those of the ND-CTRL group

The results were consistent for the majority of the 26 observed parameters, proving that the animal model was successfully induced after 16 weeks. For the remainder of the study, the trend of results between the different doses of the WJ-MSC groups did not deviate from one another but remained significantly different (*P* < 0.05) from the ND-CTRL. This finding was especially true for physical measurement, cardiovascular function tests and whole-body compositions, as shown in Fig. 1.

**Fig. 2:**
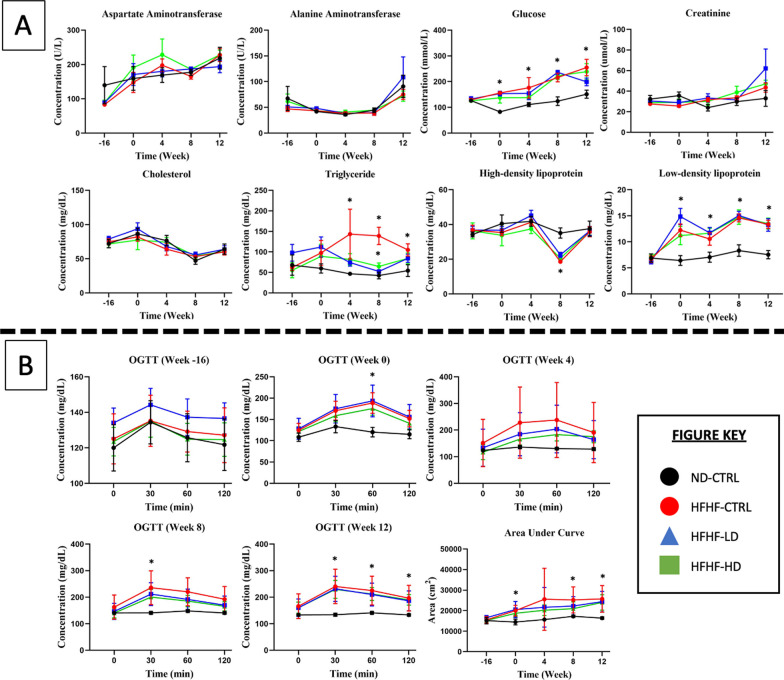
**A** Blood serum biochemistry and **B** oral glucose tolerance test performed at weeks − 16, 0, 4, 8 and 12 for the treatment (n = 8) and control (CTRL, n = 6) animals in HFHF diet or ND diet groups. Symbols: (#) indicates that the HFHF group’s data are statistically significantly different (*P* < 0.05) compared with those of the ND-CTRL group

In Fig. 2, the serum biochemistry result showed no discernible pattern between receiving the diet or WJ-MSC treatment. The OGTT amongst the HFHF-diet groups did not differ, and no explicit changes were observed even after treatment with WJ-MSC or the vehicle solution. At week 0, the insulin response for the three HFHF diet groups to glucose bolus was delayed until 60 min compared with that for the ND-CTRL group, which reduced after 30 min. During week 4, the standard deviation values drastically varied for LD and HD WJ-MSC groups, suggesting that several animals benefited from receiving WJ-MSCs. By weeks 8 and 12, the insulin response improved in all HFHF diet groups because the glucose concentration reduced after 30 min. The calculated AUC presented a large standard deviation for all HFHF diet groups before treatment and later stabilised at week 8. By week 12, the deteriorated condition returned because the treated animals had statistically significant differences (*P* < 0.05) with the ND-CTRL group.

### Subcluster analysis revealed the masked effects of WJ-MSC

As shown in Table 2, comparison of the individual results of the treated animals (n = 6 – 8/group) with those of the HFHF-CTRL group (n = 8, Appendix 1) revealed the suspected masked effects of WJ-MSCs. Prior to these results, the largest MetS effect was increased hemodynamic indices (SBP, DBP and MAP). Subsequently, the number of animals in the HFHF-HD group that showed improvement in SBP, DBP and MAP results at week 4 were 6/8 (*P* < 0.01), 3/8 (*P* < 0.05) and 1/8 (*P* < 0.05), respectively. At week 8, SBP and DBP were found to be reduced (*P* < 0.05) in 1/8 and 3/8 of the animals only. By the end of week 12, SBP and DBP decreased (*P* < 0.05) in 2/8 of the affected animals. The HFHF-LD group had a similar progression to the HFHF-HD group but only showed decreased (*P* < 0.05) SBP and DBP at week 4 in 2/8 and 1/8 of MetS animals, respectively, and at week 8 in 1/8 and 3/8, respectively. Only SBP was corrected for 1/8 of the animals until week 12.

**Table 2:** Frequency of improved symptoms in MetS animals after MSC or saline treatment at different doses throughout the study period of 12 weeks

MetS risk factors	Symptoms	Parameters observed	Frequency of improved symptoms after WJ-MSCs (n = 8/group)					
			Week 4		Week 8		Week 12	
			LD	HD	LD	HD	LD	HD
Obesity	Excess weight and fat	BW	1/6	1/6	–/6	–/6	–/6	–/6
		Abd. Circ	–/6	–/6	–/6	–/6	–/6	–/6
		BMI	–/6	–/6	–/6	–/6	–/6	–/6
		Fat Mass	–/6	–/6	–/6	–/6	–/6	–/6
		% Fat	–/6	–/6	–/6	–/6	–/6	–/6
	Lipid metabolism	TGL	–/5	–/5	–/5	–/5	–/5	–/5
		CHOL	–/2	–/3	–/2	–/3	–/2	–/3
		LDL	–/8	–/8	–/8	–/8	–/8	–/8
		HDL	–/1	–/1	–/1	–/1	–/1	–/1
	Hyperphagia	Food intake	–/8	–/8	–/8	–/8	–/8	–/8
		Caloric intake	–/8	–/8	–/8	–/8	–/8	–/8
Diabetes	Severe weight loss	BW	1/1	1/2	–/2	1/2	–/2	–/2
		Lean mass	–/2	2/2	–/2	1/2	–/2	–/2
	Hyperglycemia	GLUC	–/8	–/8	–/8	1/8	–/8	–/8
	Insulin resistance	OGTT (AUC)	1/8	1/8	1/8	2/8	1/8	1/8
	Dehydration	Water intake	–/6	–/7	–/6	–/7	–/6	–/7
Cardiovascular diseases	Hypertension	SBP	2/8	6/8	1/8	1/8	1/8	2/8
		DBP	1/8	3/8	–/8	3/8	–/8	2/8
		MAP	–/8	1/8	–/8	–/8	–/8	–/8
	Poor blood flow	Heart rate	–/8	–/8	–/8	–/8	–/8	–/8
		Blood flow	–/8	–/8	–/8	–/8	–/8	–/8
		Blood volume	–/8	–/8	–/8	–/8	–/8	–/8
Fatty liver disorder	Hepatic damage	AST	–/2	–/3	–/2	–/3	–/2	–/3
		ALT	–/2	–/3	–/2	–/3	–/2	–/3
Chronic kidney disease	Poor excretion	CREAT	–/5	–/5	–/5	–/5	–/5	–/5
Bone disease	Osteo-degeneration	BMC	–/1	1/2	–/2	1/2	–/1	–/2
		BMD	–/3	1/4	–/3	–/4	–/3	–/4

Symbols: (–) indicates no significant improvements by comparing with the range of results of HFHF-CTRL at each week (Appendix 2)

The categorised parameters for obesity, such as increased body weight and fat mass, were corrected in 1/6 of the affected animals for HFHF-LD and HFHF-HD groups during week 4 but did not remain thereafter. Diabetic-related parameters had notable improvements. The severe weight loss was resolved (*P* < 0.05) in animals from the HFHF-LD (1/1) and HFHF-HD groups (1/2). Only the effects from a larger dose of WJ-MSCs prevented weight loss until week 8. Alternatively, the lean mass of the HFHF-HD group was higher (*P* < 0.05) than that of the HFHF-CTRL group in weeks 4 and 8. Despite this finding, the primary indicator of diabetes is hyperglycaemia. According to the results, only 1/8 of the HFHF-HD animals had an improved glycaemic index (*P* < 0.05) at week 8. Oddly, no prior recovery was seen for this during week 4. A similar result was found from OGTT (AUC), where 1/8 of the HFHF-LD animals recovered between weeks 4 and 12. Moreover, 1/8, 2/8 and 1/8 of the HFHF-HD animals overcame hyperglycaemia (*P* < 0.05) during weeks 4, 8 and 12, respectively. Lastly, the BMC and BMD scores in the HFHF-HD group increased only in ½ and 1/4, respectively. Only the BMD score was sustained until week 8, extending the recovery of ½ of the affected animals.

### Post-mortem analysis of histopathology showed the protective effect of WJ-MSCs

**Fig. 3:**
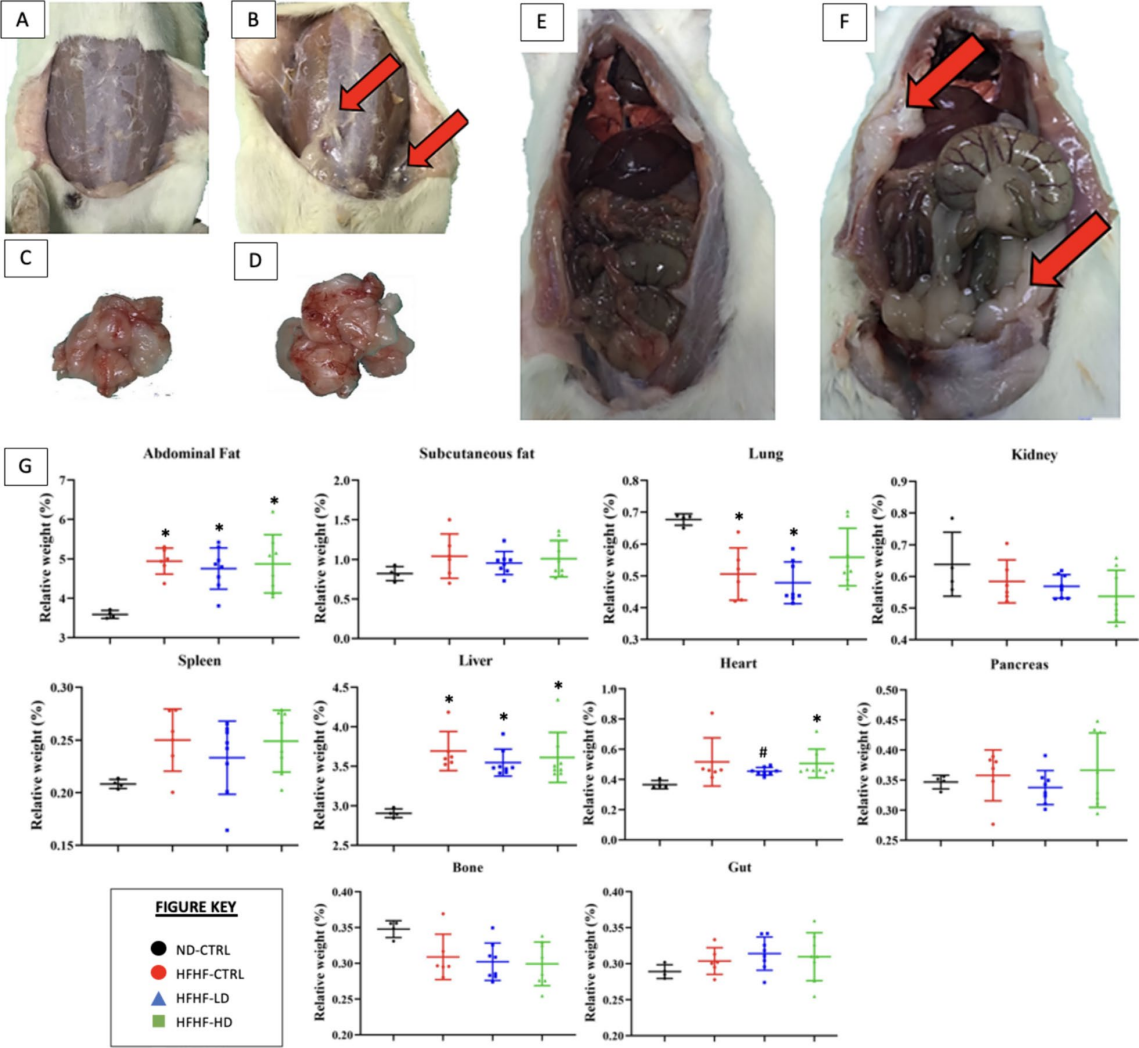
**A**–**F** necropsy and **G** relative weight of organs (n = 6–8) performed at the end of study (week 12). Images A, C and E represent the control (CTRL) healthy animals. Images B, D and F represent the typical MetS animals after induction of MetS via HFHF diet. Red arrows symbolise the deposition of fats on or around the organ. Symbols: (*) indicates that the HFHF group’s data are statistically significantly different (*P* < 0.05) compared with those of the ND-CTRL group

As shown in Fig. 3, the necropsy revealed significant deposition of fat between the subcutaneous and muscular layers in the abdomen. However, the relative weight of the organs was statistically significant (*P* < 0.05) for abdominal fat and liver only. Despite the hypertrophied heart, the relative weight was not statistically different as compensated by the increased body weight of the affected animals.

**Fig. 4:**
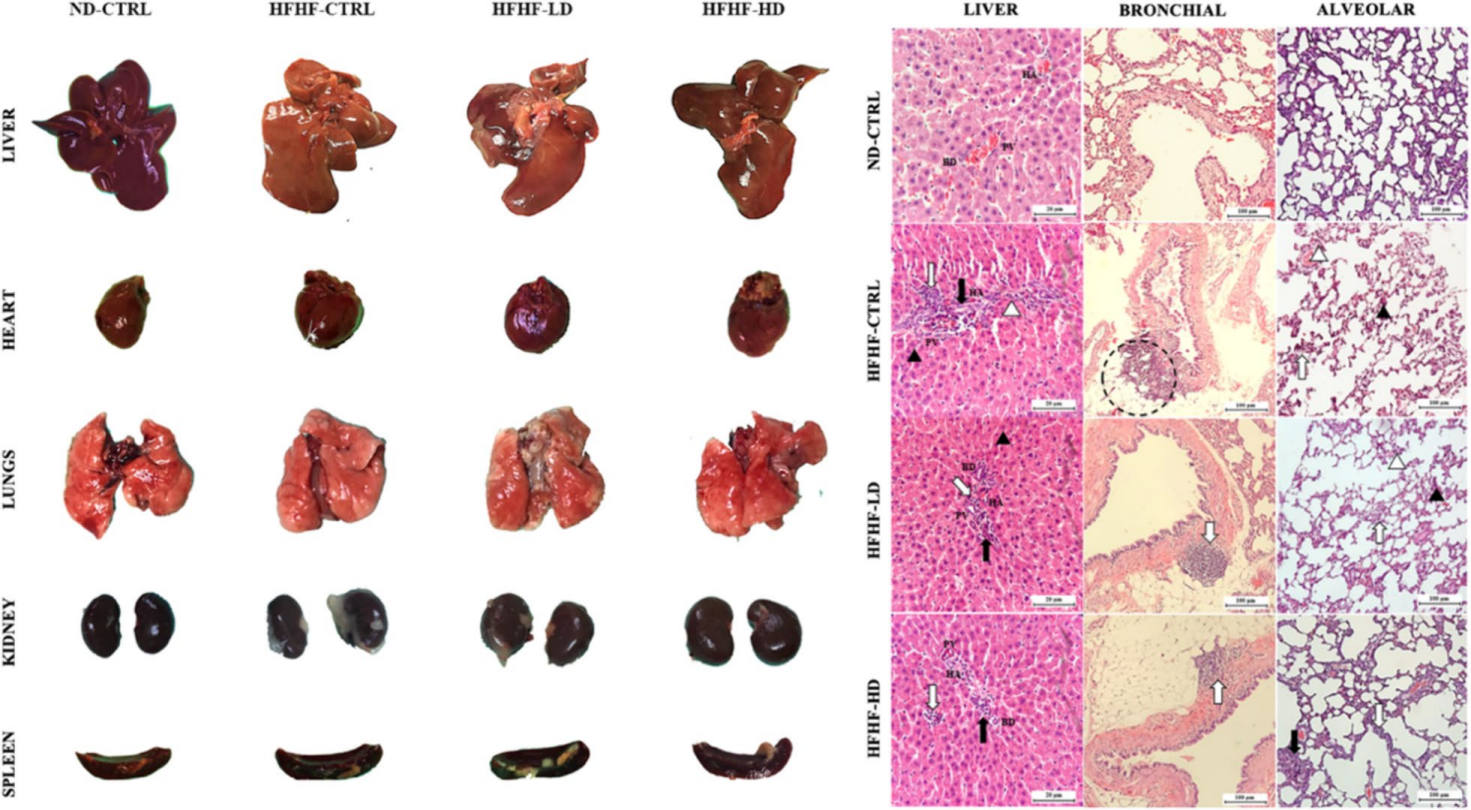
Physical observation of the harvested organs that include liver, heart, lungs, kidneys and spleen (chronological order). H&E-stained sections of the liver and lung (bronchial and alveolar) were captured at 10× and 40× magnifications with their respective scale bars (20 or 100 µm). The size and dimensions of the cropped images of the organs are not accurate representatives

In Fig. 4, the liver presented clinically significant differences as a gradient from healthy to severe inflammation in the ND-CTRL, HFHF-HD, HFHF-LD and HFHF-CTRL groups. Signs of liver cirrhosis and fatty liver were identified by the degree of ‘*yellow*’ complexion from fatty deposits and the physically defined lobes as enlarged or round. The hearts of the HFHF animals were hypertrophied, as indicated by the enlarged and round ventricles. The lungs of HFHF-CTRL and HFHF-LD animals were pale, with mild inflammation, minor lesions and pulmonary blebs (white spotting). Conversely, the lungs of the HFHF-HD group had brighter red complexion with hints of minor inflammation (potential recovery) that were comparably similar to those of the healthy animals. All the lungs were unperforated, as determined by the ability to stay afloat in a saline-filled glass beaker (data not available). The kidneys and spleens of the HFHF groups appeared to be discoloured or pale compared with those of the ND-CTRL group. No differences were observed for the bone, gut and pancreas of the animals.

The H&E images of the HFHF-CTRL group’s livers revealed major cellular hyperplasia in the surrounding parenchyma (hepatocyte cells) near the portal triads. The expanded cells constrict the interstitial spaces (sinusoidal spaces), leading to the loss or displacement of local Kupffer cells. The portal triad unit showed severe infiltration of leukocytes, necrosis and developed fibrous septum. Thus, the structures were disfigured, causing notable subunits, such as the bile duct, hepatic artery and portal vein, to be unidentifiable from severe vascular congestions. The HFHF-LD group showed similar but milder inflammation in the liver. Conversely, the HFHF-HD group preserved its microanatomical structures, with a minor leukocyte infiltration. However, the recovery may be transient or limited because a mass of leukocytes remained visible. The bronchial and alveolar sections of the lungs from the HFHF-CTRL group presented significant tissue necrosis and enhanced inflammatory status akin to the liver. A visible rupture of the bronchial wall can be observed, and it was likely a product of chronic accumulation of inflammatory damage. Meanwhile, diffused alveolar damage was manifested from the pronounced interstitial lymphocytic infiltrate into the alveolar septa. The same histopathology was seen in the lungs of the HFHF-LD group, but physical structures were more discernible. Lastly, the lungs of the HFHF-HD group were scored nearer to those of the ND-CTRL group than either the HFHF-CTRL or LD groups. No leukocyte invasion into the endothelial sections was found. However, the alveolar sacs were noticeably larger than in the other groups, which may signify the onset of respiratory stress from cardiovascular complications. No abnormalities were identified in the spleen or kidneys despite the paleness or discoloration mentioned above (Appendix 3).

### Immunomodulatory benefits of WJ-MSCs followed subcluster and histopathology results

In addition to serum biochemistry, serum inflammation-associated proteins and cytokines were analysed to study the immunomodulatory properties of WJ-MSCs. The HFHF-CTRL group expressed the most significant changes at week 12 compared with those at week 0, which were seen through IL-1α (3.3-fold), IL-1β (0.63-fold), IL-4 (0.47-fold) and MCP-1 (1.9-fold). The HFHF-LD group showed briefly increased IL-4 (0.63-fold) and MCP-1 (1.5-fold) expression levels after 12 weeks. The HFHF-HD group results were significant for IL-1β (0.61-fold) only. Lastly, the ND-CTRL group had no significant changes for most parameters but showed decreased IL-1a (0.47-fold) and IL-2 (0.41-fold) expression levels compared with week 0. IL-1β, IL-2 and IL-4 had no identifiable trend in all groups. Throughout the study, the pro-inflammatory cytokines were highly expressed in the HFHF groups versus the ND group. No significant alterations were seen after the administration of WJ-MSCs.

The serum CRP produced the same inconsistencies with the serum biochemistry from Fig. 3 in “Animals” section. During week 0, all HFHF groups had significantly increased (*P* < 0.05) CRP levels compared with the ND-CTRL group. By week 4, the animals infused with a higher dose of WJ-MSCs showed a major reduction (*P* < 0.05, intra-group comparison). The groups that received LD or vehicle solution did not change for the rest of the study. At week 8, the reduced CRP levels of the HFHF-HD were not reproduced with the same magnitude as they had during weeks 0 − 4 but remained similar to the healthy animal group. By the end of the study, the CRP concentration increased, matching those of the other HFHF groups.

## Discussion

Based on the results presented above, the overall data showed that no significant changes occurred after treatment of WJ-MSC. An unexpected consequence was the manifestation of different subclusters of MetS. Different combinations of MetS hallmarks occurred from the diet, mirroring the diverse pathways of MetS to develop any of the metabolic-associated diseases mentioned before, such as the obesity subpopulation inferred by the increased body weight and lipid markers in this study (Appendix 1). A significant number of animals (n = 19) expectedly demonstrated an increase in body weight (highest at 600.6 g), whereas few (n = 5) experienced severe weight loss (lowest at 332.4 g) paired with visible signs of morbidity such as heavy perspiration, fatigue, accumulated porphyrin and decreased food and water intake. The underweight animals were below the weight range of the healthy untreated animals. This grouped data resulted in no statistically significant differences because the ND group (n = 6) and HFHF group (n = 24) had body weights of 410.65 ± 35.27 and 475 ± 66.07 g, respectively. Biased data between these subpopulations became increasingly distinct as the study progressed. The underweight animals co-expressed highest GLUC reading (11.0 mmol/L or 198 mg/dL), whereas the obesogenic animals had higher lipid-associated markers. Based on the symptoms presented, the smaller group were hypothesised to be pre-diabetic likely through acquired insulin resistance [30–35].

Previous studies have encountered different subclusters of MetS specifying their methodology and controlled conditions [36–38]. For example, Rozendaal et al. (2018) mapped the disease progression of MetS through in-vivo and in-silico models [39]. Although successful, the authors expressed concerns over their dynamic study model producing the unexpected outcome of two MetS phenotypes. In retrospect, this study’s animal model may have been selective towards obesity-focused MetS by excluding other subclusters. However, the fulfilment of the minimal criteria for MetS in other animals does not justify excluding potentially valuable data. Moreover, the inclusion of different metabolic diseases, obesity, diabetes, CVDs or MAFLD could benefit the scope of this study. However, the manifestation of polarising data in the same group was unprecedented. Besides the weight of the animals, several other parameters responded differently to HFHF diet, potentially negating the effects of infused WJ-MSCs. At least ≥ 10 of the 26 test parameters had mixed response (higher or lower) compared with the reference data from the healthy animals. For example, the BMC and BMD values may have been overestimated due to the obesity paradox. Increased body weight exerts a mechanical load that drives the increase in BMC and BMD in rats. However, adiposity contributes to increased chronic inflammation (e.g. SOD or MDA), which may cause bone deterioration [38, 39]. The animals data were individually analysed versus the reference data from the untreated (HFHF-CTRL) group to circumvent this issue, corresponding to the same study period (Appendix 2). Based on the evidence presented in Sect. 2.2, WJ-MSCs were unable to treat MetS as a conventional means of a cure. In Figs. 1 and 2, WJ-MSCs may have attenuated the effects of MetS. However, not all the animals responded similarly to the treatment.

In Sect. 2.3, WJ-MSCs were hypothesised to be responsible for delaying the metabolic derangement of MetS animals. The masked results of WJ-MSCs were obtained after each animal’s data (n = 8/group) were screened for 26 parameters during each study period (weeks 0, 4, 8 and 12; Table 2). WJ-MSCs positively affected the increased hemodynamic indices. This finding is consistent with many successful MSC applications for cardiopulmonary complications [42–46]. For example, Alencar et al. (2018) outlined the benefits of MSCs in improving the symptoms of pulmonary arterial hypertension [47]. The treatment enabled cardiac cell repolarisation, preventing further cell apoptosis and fibrosis. It also reduced muscle stiffness and the rigidity of vascular endothelial walls, restoring normal blood circulation. Whilst the mortality of diabetic animals was undetermined, major improvements were noted in the animal’s health (signs of morbidity) through returned weight and normal behaviour. The physical wellbeing of the animals did not deteriorate until weeks 4 and 8 for the LD and HD WJ-MSC groups, respectively, implying the importance of dose selection.

The necropsy and subsequent histological staining of organs were consistent with the subgroup analysis above. The liver, lungs, and heart had enhanced pathological outcome, as shown by the reduced inflammation and hypertrophy, following the administration of HD WJ-MSCs, similar to previous studies [48–50]. Given that the HFHF diet was given ad libitum until the end of the study, the effects of WJ-MSCs may have been reduced or negated. During MetS induction, only seven (29.1%) and five (20.1%) of the 24 HFHF diet-assigned animals had increased AST and ALT, respectively (Appendix 1). Even after WJ-MSC treatment, the levels remained unchanged. These findings were justified in previous literature that inferred that liver degeneration could be masked by ‘*normal*’ liver biomarkers [33, 47]. In the present study, the authors speculated that the unchanged liver biomarkers were a byproduct of the continued HFHF diet. However, the histological images of the organs in the HFHF-HD group were less inflamed and structurally similar to those of the healthy animals and superior to those of the untreated and LD groups.

**Fig. 5:**
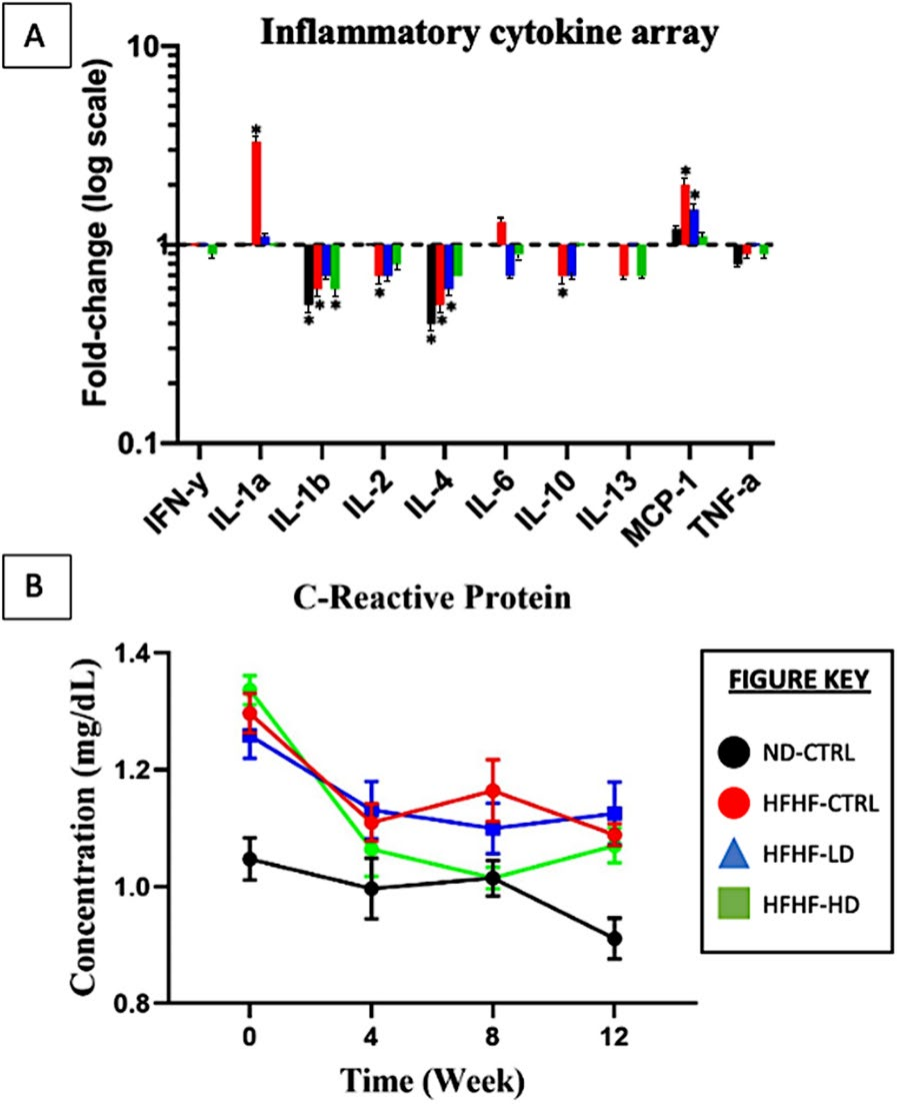
**A** Pro-inflammatory cytokines (IFN-γ, IL-1α, IL-1β, IL-2, IL-6, MCP-1 and TNF-α) and anti-inflammatory cytokines (IL-4, IL-10 and IL-13) expressed as fold-change (logarithmic scale) compared between the results (n = 4/group) from weeks 0 and 12. **B** C-reactive protein (n = 6) measured at weeks 0, 4, 8 and 12 for all animal groups. Symbol (*) indicates statistically significant change at ≥ 1.5-fold increase or ≥ 0.65-fold decrease

A major characteristic of MetS is persistent and damaging inflammation [51, 52]. Hence, addressing the inflammation could supersede the relevancy of all other variables of MetS measured thus far. Based on Fig. 5, the CRP levels at weeks 4 and 8 significantly reduced (*P* < 0.05) compared with those at week 0 for the HFHF-HD group but not for the HFHF-LD nor HFHF-CTRL group. Oddly, the CRP of the HFHF-CTRL group drastically reduced, deviating from the expectations. Although CRP is known to increase inflammation, chronic developments, such as liver dysfunction or specific diseases (e.g. rheumatoid arthritis or lupus), could manifest normal-to-low serum levels [53–55]. Similar to serum biochemistry in Fig. 2A, many inconsistencies within the inflammation array may be attributed to the limited samples and serum conditions. On the basis of the manufacturer’s instructions, a fold-change (cytokine concentration based on log–log regression standard curves) to stabilise the data with extreme variances was applied [56]. The inflammatory cytokine results between HFHF-LD and HFHF-HD had no significant changes after treatment, reaffirming the lack of WJ-MSC regenerative effect or overwhelming persistence of MetS inflammation.

Concerningly, all serum-associated parameters performed through a standard statistical analysis were difficult to interpret. Although it is a major component of toxicological studies, the validity of serum analysis is often hindered by insufficient volume for replication, sample haemolysis, batch-to-batch variation, contamination and more [57–59]. Due to movement restrictions during the recent pandemic of 2021, the HFHF diet was forcibly extended from 12 to 16 weeks. This extension strengthened the characteristics of MetS and considerably eased the identification of subclusters [60]. However, whether the delayed treatment damaged the salvageability of the metabolic health in these animals was contemplated. The WJ-MSCs could have more explicit effects at sub-chronic periods of MetS as originally intended at 12 weeks. The continuous deterioration of MetS symptoms via HFHF diet supplementation has been a universal theme in this study, proving that the antagonists of poor dietary choices and sedentary lifestyles must be equally considered [38]. Nutritional studies have successfully demonstrated that dietary correction can reverse MetS symptoms [61–64]. The same was determined for physical exercises [65–68]. However, the removal of the HFHF diet or the introduction of any physical stimulus could conflict with the research objective of determining the potency of the cell therapy. Another potential modification is to implement multiple doses of the stem cells. Most studies have adopted multiple doses at a fixed period or intermittent intervals [67, 69, 70]. The reason for repeatedly introducing stem cells into patients is to supplement a continuous reparative effect. Based on a previous systematic review on the biodistribution of MSCs, the cells can survive for up to 72 h in-vivo, and the effects persisted for a maximum of 14 days [71]. This study’s method of systemic delivery may have also suffered from poor efficacy due to the accumulated loss of cells via lysis, impenetrability of the membrane, misroute or excretion [72–74]. However, the availability of systemic administration could benefit from difficult-to-reach areas [75].

In the context of cell passage, passages 1 − 4 are conventionally used to preserve MSC nascent qualities and prevent accumulation of senescent or genetic variation from long-term culture. However, previous evidence cited the safety and efficacy of cells from passages ≥ 5 and in similar Sprague Dawley models [76–78]. Wang et al. (2021) implied that the differences between passages 4 and 6 were not as significant as those amongst longer passages 8, 10 and 12, thus justifying the preferences of this study’s design [79]. Furthermore, the usage of cells from later passages for the experimental studies enabled the production of a sufficient number of cells to ensure the same batches of cells were used from the in-vitro characterisation phase to the safety and efficacy studies in the animal model study phase.

Another valid argument is the obstruction of transplanted human WJ-MSCs in the animal model. The authors foresaw that the effects of the cell therapy could be limited by unknown xenogeneic components [80–83]. An accurate allogeneic transplant (e.g. rodent-derived cells into animal model or human-derived cells into patients) may have produced different efficacy outcomes. However, the importance of testing medicinal products in pre-clinical models is majorly driven by safety [84]. Regardless of animal derivative studies, the succeeding phase (current study) for utilising human-derived cells must be tested using pre-clinical models before humans. Based on the previous and current studies, neither the healthy nor diseased animals responded adversely to the administered WJ-MSCs. The prospects for positive outcomes or recovery by stem cell therapy is promising.

This study has limitations. Firstly, one of the several issues that occurred was the insufficient serum volume from the blood, accompanied by minor-to-significant haemolysis. Due to the small size of the animals, blood was difficult to acquire, and therefore, the results were not fully representative of their metabolic health and inflammatory status. Secondly, the type of MetS animal models generated from diet-induced methods must be wholly considered early or prior into the study. The subcluster analysis was only a temporary solution. Thirdly, a single dose of MSC for 3 months could not elicit a significant reaction in the diseased animal model, much less in 2 weeks [71]. Lastly, the histological analyses, which had significant and relevant findings but were limited by the study’s resources, should have been further expanded. A suggestion for future studies is incorporating immunohistochemistry because it could provide valuable information on the treatment’s efficacy.

For future consideration, the authors proposed to include more than one dose at fixed intervals to explore the continuous effects of WJ-MSCs. A previous systematic review highlighted many recent studies that adopted multiple doses without any adverse outcomes [70]. Moreover, the heterogeneity of MetS is too diverse and complex to be compressed into a single study [38]. However, it may be compensated by adopting a larger sample size or additional inclusion and exclusion criteria to define the type of MetS studied [85]. Furthermore, recent efforts to study the functional, small extracellular vesicles from MSCs have proposed better clinical outcomes than cell therapy for future applications [24]. Consequently, MSCs may be better as support for other medications because they are well-positioned to stimulate regeneration and regulate inflammation simultaneously.

## Conclusion

The WJ-MSCs were not able to correct the symptoms of MetS. However, further analysis showed that the continuous worsening state of the animals and subcluster profiles may have overwhelmed the effects of WJ-MSCs. Based on the new evidence presented, the WJ-MSCs exert a protective effect seen through delayed metabolic deterioration in vivo. The treatment effects were observed to be in a dose-dependent manner. The organs (lungs and liver) showed obvious preservation from receiving WJ-MSCs. The major MetS subclusters of cardiovascular impairments showed early improvements at week 4, which were sustained through week 8 through receiving a higher dose of the cells. Incidentally, CVD is the largest MetS comorbidity and the highest global cause of death by noncommunicable diseases. All the recovery made possible through WJ-MSC therapy was collectively reversed by week 12. Thus, this single infusion of WJ-MSCs was able to exert its regenerative and immunomodulatory effects for up to a maximum of 8 weeks. By securing the in-vivo safety in healthy and diseased animals, multiple doses of WJ-MSCs or co-therapy with pharmaceutical interventions, dietary changes, exercise regimes, and more are suggested for future exploration. Furthermore, the selection of specific MetS subclusters versus the whole MetS must be considered to validate any therapeutic efficacies in the future.

## Figures and Tables

**Fig. 6:**
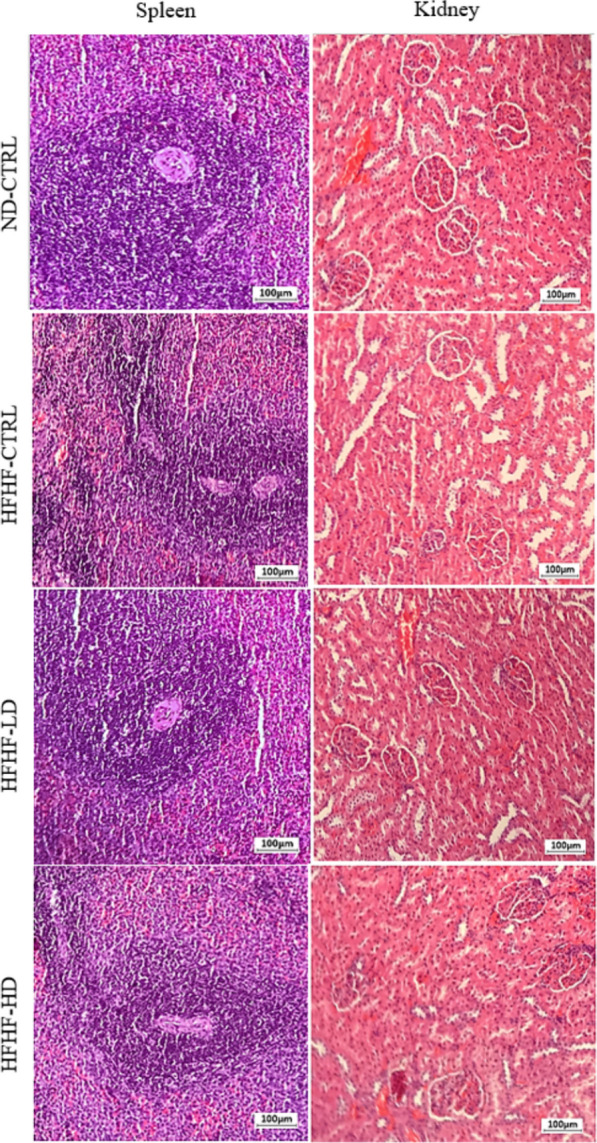
H&E images of kidney and spleen for all treatment groups (top to bottom rows): ND-CTRL, HFHF-CTRL, HFHF-LD and HFHF-HD. Images were captured at 10 × magnification with scale bar (100 µm)

## Data Availability

Data will be made available upon reasonable request.

## Appendix 1

See Table

**Table 3:** Frequency of affected parameter for each individual animal given HFHF diet after 16 weeks of diet program

MetS Parameters	Healthy animals (n = 5)	Induced MetS animals (n = 24)		
	Range	Higher	Lower	No change
Body weight (g)	371.0–441.6	17	4	3
Body length (cm)	24.05–24.63	–	–	24
Abdominal Circumference (cm^2^)	16.86–20.86	17	–	7
Body Mass Index (g/cm^2^)	0.620–0.720	20	1	3
Food Intake (g)	21.08–25.08	–	24	–
Water Intake (mL)	26.13–32.03	17	2	5
Caloric intake (kCal)	294.5–350.5	24	–	–
Systolic Pressure (mmHg)	102.8–123.6	24	–	–
Diastolic Pressure (mmHg)	83.15–95.05	24	–	–
Mean Arterial Pressure (mmHg)	74.42–87.68	24	–	–
Heart Rate (beats/min)	361.7–423.1	24	–	–
Blood Flow (mL/min)	17.00–20.70	–	24	–
Blood Volume (mL)	23.75–28.25	18	3	3
Bone mineral content (g)	15.14–15.40	–	4	20
Bone mineral density (g/cm^2^)	0.195–0.199	11	8	5
Fat Mass (g)	51.93–64.03	19	3	2
Lean Mass (g)	300.4–355.7	17	4	3
Body fat percentage (%)	14.57–15.43	22	2	–
Aspartate aminotransferase (U/L)	90.30–228.8	7	3	14
Alanine transaminase (U/L)	34.60–49.00	5	1	18
Glucose (mg/dL)	79.80–85.80	24	–	–
Creatinine (µmol/L)	28.34–42.86	–	15	9
Cholesterol (mg/dL)	69.98–102.8	7	5	12
Triglyceride (mg/dL)	37.64–82.00	12	6	6
Low-density lipoprotein (mg/dL)	4.550–8.290	24	–	–
High-density lipoprotein (mg/dL)	30.45–50.49	2	1	21

^3^.

## Appendix 2

See Table 4.

**Table 4:** Range of HFHF-CTRL at each study period used to compare with data from WJ-MSC treated groups

MetS Parameters	Range of results of HFHF-CTRL (n = 8)			
	0	4	8	12
Body weight (g)	452 ± 77.4	482 ± 92.3	502 ± 94.0	508 ± 99.3
Body length (cm)	24.1 ± 0.75	25.3 ± 1.02	26.0 ± 1.16	26.5 ± 0.82
Abdominal Circumference (cm^2^)	21.6 ± 2.61	23.1 ± 2.77	23.7 ± 3.00	24.3 ± 3.32
Body Mass Index (g/cm^2^)	0.81 ± 0.10	0.74 ± 0.11	0.73 ± 0.10	0.71 ± 0.11
Food Intake (g)	15.7 ± 2.46	17.3 ± 3.52	20.4 ± 4.79	23.4 ± 6.77
Water Intake (mL)	32.0 ± 2.54	38.8 ± 6.35	39.7 ± 6.11	40.0 ± 8.16
Caloric intake (kCal)	519 ± 65.9	590 ± 108	664 ± 138	733 ± 191
Systolic Pressure (mmHg)	144 ± 11.7	141 ± 8.62	142 ± 11.4	142 ± 6.69
Diastolic Pressure (mmHg)	117 ± 4.34	118 ± 6.10	117 ± 7.23	118 ± 3.77
Mean Arterial Pressure (mmHg)	106 ± 7.32	106 ± 9.56	106 ± 12.0	107 ± 5.67
Heart Rate (beats/min)	537 ± 30.6	531 ± 28.0	534 ± 33.7	531 ± 18.7
Blood Flow (mL/min)	6.05 ± 0.66	6.45 ± 0.67	6.65 ± 0.67	6.81 ± 1.09
Blood Volume (mL)	30.7 ± 4.97	31.3 ± 6.02	32.8 ± 6.28	33.0 ± 6.57
Bone mineral content	17.2 ± 2.09	17.0 ± 2.10	17.1 ± 2.27	17.1 ± 2.20
Bone mineral density (g/cm^2^)	0.190 ± 0.009	0.191 ± 0.008	0.190 ± 0.009	0.199 ± 0.008
Fat Mass (g)	90.1 ± 26.9	95.3 ± 30.8	100 ± 31.3	106 ± 32.4
Lean Mass (g)	359 ± 55.4	367 ± 66.7	383 ± 70.3	398 ± 78.0
Body fat percentage (%)	19.5 ± 2.78	19.9 ± 3.46	20.2 ± 3.40	20.4 ± 3.60
Aspartate aminotransferase (U/L)	147 ± 65.8	197 ± 41.6	165 ± 16.4	227 ± 41.8
Alanine transaminase (U/L)	43.5 ± 3.86	38.5 ± 6.64	38.9 ± 8.83	75.1 ± 21.5
Glucose (mg/dL)	155 ± 8.15	176 ± 89.2	216 ± 33.6	254 ± 71.2
Creatinine (µmol/L)	25.5 ± 2.51	31.6 ± 4.50	33.6 ± 3.72	43.5 ± 10.0
Cholesterol (mg/dL)	81.9 ± 13.9	63.8 ± 19.1	53.2 ± 4.62	60.1 ± 10.5
Triglyceride (mg/dL)	97.6 ± 67.9	143 ± 135	139 ± 42.0	104 ± 33.2
Low-density lipoprotein (mg/dL)	12.2 ± 2.65	10.6 ± 2.66	14.6 ± 1.52	13.4 ± 1.75
High-density lipoprotein (mg/dL)	35.4 ± 3.97	41.3 ± 3.74	18.6 ± 1.41	35.8 ± 6.43

## Appendix 3

See Fig. 6.

## Abbreviations

BMC: Bone mineral content
BMD: Bone mineral density
CREAT: Creatinine
CRP: C-reactive protein
CTRL: Control
DBP: Diastolic blood pressure
GLUC: Glucose
HD: High dose
HFHF: High-fat high-fructose
LD: Low dose
MAP: Mean arterial pressure
MCP-1: Monocyte chemoattractant protein-1
MetS: Metabolic syndrome
ND: Normal diet
OGTT: Oral glucose tolerance test
SBP: Systolic blood pressure
WJ-MSC: Wharton’s jelly-derived mesenchymal stem cell
